# LC–MS-Based Urine Metabolomics Analysis for the Diagnosis and Monitoring of Medulloblastoma

**DOI:** 10.3389/fonc.2022.949513

**Published:** 2022-07-22

**Authors:** Xiaoyan Liu, Jing Li, Xiaolei Hao, Haidan Sun, Yang Zhang, Liwei Zhang, Lulu Jia, Yongji Tian, Wei Sun

**Affiliations:** ^1^ Core Instrument Facility, Institute of Basic Medical Sciences, Chinese Academy of Medical Sciences, School of Basic Medicine, Peking Union Medical College, Beijing, China; ^2^ Department of Neurosurgery, China National Clinical Research Center for Neurological Diseases, Beijing Tiantan Hospital, Capital Medical University, Beijing, China; ^3^ Department of Pharmacy, Clinical Research Center, Beijing Children’s Hospital, Capital Medical University, National Center for Children’s Health, Beijing, China

**Keywords:** medulloblastoma, urine, metabolomics, biomarker, tetrahydrocortisone, cortolone

## Abstract

Medulloblastoma (MB) is the most common type of brain cancer in pediatric patients. Body fluid biomarkers will be helpful for clinical diagnosis and treatment. In this study, liquid chromatography–mass spectrometry (LC–MS)-based metabolomics was used to identify specific urine metabolites of MB in a cohort, including 118 healthy controls, 111 MB patients, 31 patients with malignant brain cancer, 51 patients with benign brain disease, 29 MB patients 1 week postsurgery and 80 MB patients 1 month postsurgery. The results showed an apparent separation for MB vs. healthy controls, MB vs. benign brain diseases, and MB vs. other malignant brain tumors, with AUCs values of 0.947/0.906, 0.900/0.873, and 0.842/0.885, respectively, in the discovery/validation group. Among all differentially identified metabolites, 4 metabolites (tetrahydrocortisone, cortolone, urothion and 20-oxo-leukotriene E4) were specific to MB. The analysis of these 4 metabolites in pre- and postoperative MB urine samples showed that their levels returned to a healthy state after the operation (especially after one month), showing the potential specificity of these metabolites for MB. Finally, the combination of two metabolites, tetrahydrocortisone and cortolone, showed diagnostic accuracy for distinguishing MB from non-MB, with an AUC value of 0.851. Our data showed that urine metabolomics might be used for MB diagnosis and monitoring.

## Introduction

Medulloblastoma (MB) is the most frequent malignant central nervous system neoplasm in children (1, 2), accounting for 8% to 10% of childhood brain tumors (3). Early detection and treatment are clearly effective methods for improving the five-year survival rate, which is up to 85%-90% for average-risk disease and up to 65% for high-risk disease (4–7). Clinically, dynamic contrast-enhanced computed tomography and magnetic resonance imaging are commonly used imaging techniques for the diagnosis of MB in most cases (8). However, differentiating benign brain diseases and malignant tumors from MB remains a clinical challenge, even when images are re-examined by experienced radiologists (9). Thus, the development of new, accurate, and noninvasive diagnostic methods will have an important impact on the clinical treatment of MB in its earliest stages and could lead to a reduction in unnecessary treatment for other brain diseases.

Metabolomics is a useful strategy for identifying potential biomarkers for disease (10). It provides links as downstream to molecular divergence occurs. Metabolites, being the end products, are more stable than mRNAs or proteins. Metabolite identification is highly informative about the functional status of the biological system, owing to their proximity to organismal phenotypes. Previous studies have shown the efficacy of metabolomics in identifying biomarkers associated with the diagnosis, prognosis and treatment of cancer (11) Recently, brain tumors have become well characterized due to large metabolic studies (12), and some studies have provided metabolomic analyses for MB using tissue samples. In 2010, Cuellar-Baena, S et al. presented metabolic profiles by NMR analysis of brain tumor tissues, including ependymoma and MB. The NMR profile of pediatric MBs showed important metabolic information, including increased membrane turnover, low neuronal viability and glycolysis alterations (13). In 2018, using NMR, Christopher D. Bennett et al. analyzed tissue metabolites to discriminate cerebellar ependymoma, MB and other brain tumors, and their study showed high phosphocholine and taurine levels in MB (9). Several studies have tried to explore MB metabolism signatures using biofluids. In 2022, Bongyong Lee et al. performed an integrated analysis of the transcriptomic, metabolomic, and lipidomic changes in the CSF of children with MB. The tricarboxylic acid cycle and total triacylglycerols were found to be significantly upregulated in MB patients. A group of omics signatures, including indicators of tumor hypoxia, was found to separate MB from normal samples (14).

Urine is an important biofluid to screen for biomarkers of brain tumors. Collection of urine is risk free and easy. In particular, urine sampling avoids the need for sedation and its associated risks, which is often required to accomplish radiographic studies in the pediatric population. Moreover, urine collection and analysis are considerably less expensive than magnetic resonance imaging. Sampling of urine could easily be done at shorter intervals than are currently practical for imaging studies, enabling earlier detection of recurrent disease (15). Several studies have attempted to discover urinary biomarkers for other brain cancers. In 2011, Moroz, J et al. analyzed the metabolic content of urine from NIH III nude mice (n = 22) before and after inoculation with human glioblastoma multiforme (GBM) cancer cells. The number of statistically significant changes in urinary metabolites was more pronounced in the tumor-bearing population than in the control animals. The results showed that metabolomics may be used as a screening tool for GBM cells grown in mice (16). Previous studies have shown that it is possible to identify urinary biomarkers of brain cancer. Therefore, the aim of this study was to utilize urine metabolomics to explore potential biomarkers for the detection of MB. This study might provide new insight into the differential diagnosis of MB from controls.

## Materials and Methods

### Patients and Sample Collection

This study was approved by the Institutional Review Board of the Institute of Basic Medical Sciences, Chinese Academy of Medical Sciences. Written informed consent was obtained from the participants before participation in this study. The cohort included 111 MB patients, 51 patients with benign brain disease, 31 patients with other malignant brain cancers, 118 healthy volunteers, 29 1-week postoperative MB patients, and 80 1-month postoperative MB patients (**Table S1**). The brain tumor patients scheduled for surgical resection were prospectively screened from the Beijing Tiantan Hospital, Capital Medical University. Only those patients aged 16 years or less who underwent radical surgery for pathologic diagnosis were recruited. Preoperative urine samples were collected immediately before surgery, while postoperative samples were collected 1-2 weeks or 1-3 months after surgery. These groups did not include subjects suffering from any acute conditions or subjects administered any drugs. The first morning urine (midstream) samples were collected at approximately 07:00 to 09:00 a.m. on an empty stomach from the patients or healthy volunteers. The urine samples were centrifuged within 6 h after collection; the supernatants were isolated, aliquoted, and stored at −80°C until analysis.

### Sample Preparation

Urine sample preparation was performed based on a previous method (17). In brief, acetonitrile (200 μL) was added to each urine sample (200 μL), and the mixture was vortexed for 30 s and centrifuged at 14,000 *×g* for 10 min. The supernatant was dried under vacuum and then reconstituted with 200 μL of 2% acetonitrile. Urinary metabolites were further separated from larger molecules using 10 kDa molecular weight cutoff ultracentrifugation filters (Millipore Amicon Ultra, MA) before being transferred to autosamplers.

The quality control (QC) sample was a pooled urine sample prepared by mixing aliquots of fifty representative samples across different analysis groups and was therefore globally representative of the whole sample set. The QC samples were injected every ten samples throughout the analytical run to provide a set of data from which the stability and repeatability of the method can be assessed.

### LC–MS Analysis

Ultra-performance LC–MS analyses of samples were conducted using a Waters ACQUITY H-class LC system coupled with an LTQ Orbitrap Velos pro mass spectrometer (Thermo Fisher Scientific, MA, USA). The detailed parameters are listed in Document S1.

### Statistical Data Analysis

Raw data files were processed by Progenesis QI (Version 2.0, Nonlinear Dynamics) software and further processed by MetaboAnalyst 3.0 (http://www.metaboanalyst.ca). The analysis included peak alignment, peak picking, normalization and peak identification. Pattern recognition analysis (principal component analysis (PCA) and orthogonal partial least squares discriminant analysis (OPLS-DA)) was carried out using SIMCA 14.0 (Umetrics, Sweden) software. The detailed parameters are listed in Document S1.

### Metabolite Annotation and Pathway Analysis

The metabolic pathways were first analyzed using MS1 features by the mummichog algorithm, which leverages the organization of metabolic networks to predict functional pathways directly from feature tables. Significantly different metabolites (adjusted p value < 0.05; VIP >1) were further determined from the exact mass composition, from the goodness of isotopic fit for the predicted molecular formula and from MS/MS fragmentation comparing hits with databases (HMDB, Massbank, METLIN, and mzCloud), thus, qualifying for annotation at MSI level II using Progenesis QI (Version 2.0, Nonlinear Dynamics, UK). For endogenous metabolites lacking a chemical formula, an accurate molecular mass was given based on the calculated isotopic features and ion adducts. Exploratory ROC analysis was performed by MetaboAnalyst 3.0. Detailed methods are listed in Document S1.

## Results

### Study Workflow

A total of 420 patients were enrolled in our study, including 118 healthy controls, 111 MB patients, 31 patients with malignant brain cancer (ependymoma, pilocytic astrocytoma, and glioblastoma) and 51 patients with benign brain disease (epilepsy and cramp). These samples were randomly divided into two cohorts, discovery and validation sets. Additionally, we enrolled 29 MB patients 1 week postsurgery and 80 MB patients 1 month postsurgery for biomarker validation. The patients were matched for age and sex among the different groups (**Table 1**, detailed data in **Table S1**).

**Table 1:** Demographics of medulloblastoma patients, brain benign disease patients, other brain malignant tumour patients and healthy controls.

Sample group		No. Males	No. Females	sum	Mean Age ± SD
Discovery group	Medulloblastoma	44	30	74	7.67 ± 3.34
	Healthy control	43	33	76	7.76 ± 2.06
	Brain malignant tumor patients	10	10	20	7.03 ± 4.27
	Brain benign disease patients	24	10	34	7.17 ± 3.81
Validation group	Medulloblastoma	22	15	37	7.37 ± 3.37
	Healthy control	24	18	42	7.78 ± 2.31
	Brain malignant tumor patients	6	5	11	8.1 ± 3.49
	Brain benign disease patients	13	4	17	7.17 ± 3.51
postsurgical validation	MB patients of a week after surgery	13	16	29	7.50 ± 3.27
	MB patients of a month after surgery	51	29	80	7.67 ± 3.34
sum		250	170	420	

**Figure 1:**
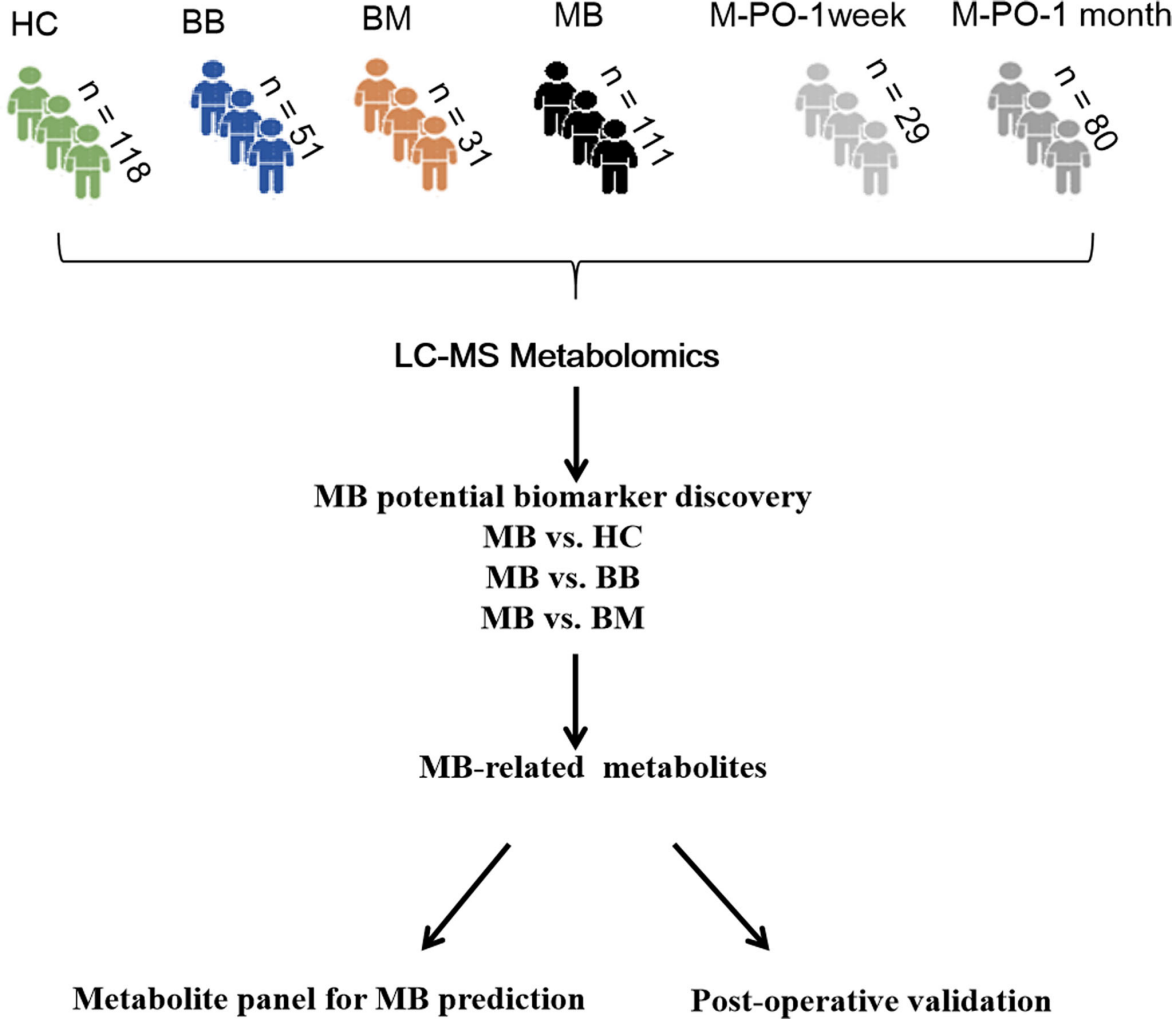
Study workflow. HC, healthy control; BB, benign brain disease; BM, other malignant brain tumor; MB, medulloblastoma; M-PO-1 week, medulloblastoma 1 month postoperatively, M-PO-1 month: medulloblastoma 1 month postoperatively.

All urine samples were analyzed by LC–MS. Comparison analysis was performed based on MB vs. healthy controls, MB vs. benign brain diseases, and MB vs. malignant brain tumors, and differential metabolites were found through a selection criterion (VIP value >1, p value < 0.05 and FC >1.5). The differential metabolites of the above three comparisons were further functionally annotated. Moreover, the differential metabolites from the above three analyses were compared to define the specific metabolites of MB. These metabolites were evaluated in MB surgery patients after one week and one month to validate the specificity. Finally, the MB diagnostic predictive model was constructed based on MS-specific metabolites. The workflow of this study is shown in **Figure 1**.

### Quality Control

All samples were randomly analyzed by LC–MS. QC is of great importance in large-scale metabolomics studies to ensure stable system performance and to avoid experimental bias. A QC standard was prepared as a pooled mixture of aliquots from all urine samples in each group. For urine metabolomics, the QC sample was injected 5 times before the analytical run and was frequently injected once every ten samples throughout the analytical run to monitor instrument stability. Tight clustering of the QC samples **(** **Figure S1** **)** indicates good consistency in the QC data.

### Distinguishing MB Patients From Healthy Controls by Urine Metabolomics

**Figure 2:**
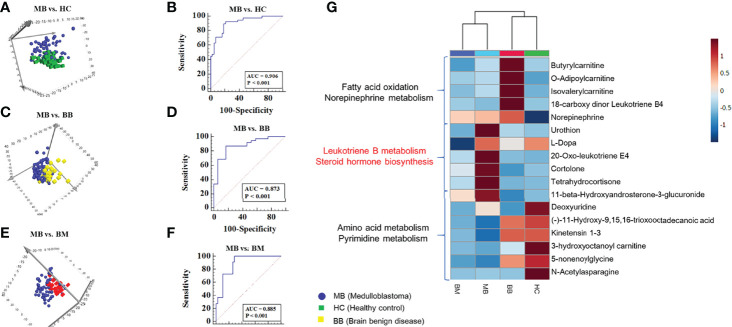
Analysis of urine metabolomics of medulloblastoma, healthy controls, benign brain diseases, and malignant brain tumors. **(A).** PCA score plot of MB and controls in the discovery group. **(B).** ROC plot of the distinction between MB and controls in the validation Group **(C).** PCA score plot of MB and benign brain diseases in the discovery Group **(D).** ROC plot of the distinction between MB and benign brain diseases in the validation Group **(E).** PCA score plot of MB and other malignant tumors in the discovery group. **(F).** ROC plot of the distinction between MB and other malignant tumors in the validation group **(G)**. Heatmap of 60 metabolites in MB, healthy controls, benign brain diseases and other malignant brain tumors.

The LC–MS-based urine metabolome from MB and healthy controls yielded 2437 spectral features. To select potential biomarkers for distinguishing MB from controls, multivariate statistical analysis models were applied. PCA showed a discrimination trend between the metabolic profiles of the MB patients and those of the healthy control subjects **(** **Figure 2A** **)**. The OPLS-DA model achieved better separation **(** **Figure S2A** **)**. One hundred permutation tests were carried out to confirm the stability and robustness of the supervised models **(** **Figure S2B** **)**. Pathway enrichment analysis using MS features by the mummichog algorithm showed the significant enrichment of several pathways, including caffeine, tryptophan, fatty acid, biopterin and urea cycle/amino group metabolisms **(** **Figure S2C** **)**.

The significantly different features were submitted to MS/MS fragmentation and Progenesis QI identification. Overall, 11 significantly different metabolites were identified (**Table S2A** **)**. The diagnostic accuracy of the differentially identified metabolites for MB and control samples was evaluated. A total of 9 metabolites had good diagnostic value with AUCs values above 0.7 **(** **Table S2B** **)**. Multivariate ROC curve-based exploratory analysis was employed to achieve a better predictive model (http://www.metaboanalyst.ca/faces/Secure/upload/RocUploadView.xhtml) using these differential metabolites. A panel consisting of tetrahydrocortisone, N-acetylasparagine, and cortolone showed the best predictive ability, with an AUC value of 0.947 in the discovery group **(** **Figure S2D**, **Table 2** **)** and 0.906 in the validation group (**Figure 2B** **) (**sensitivity: 0.892 and specificity: 0.810).

**Table 2:** AUC values of panels for MB and other groups distinction on urine metabolomics.

	The AUC of discovery group	The AUC of validation group	sensitivity	specificity:
MB vs. healthy control(a)	0.947	0.906	0.892	0.810
MB vs. brain benign diseases (b)	0.900	0.873	0.892	0.882
MB vs. other malignant tumours (c)	0.842	0.885	1	0.702
MB vs. non-MB(d)	0.888	0.801	0.908	0.757

(a) panel: Tetrahydrocortisone; Cortolone; N-Acetylasparagine.

(b) panel: Tetrahydrocortisone; Cortolone; 18-carboxy dinor Leukotriene B4.

(c) panel: Tetrahydrocortisone; Cortolone; L-Dopa; 20-Oxo-leukotriene E4.

(d) panel: Tetrahydrocortisone; Cortolone.

### Distinguishing MB From Benign Brain Diseases and Other Malignant Brain Tumors

The urine metabolome from MB and benign brain disease samples was analyzed using multiple statistical methods similar to those described above. The PCA score plot and the OPLS-DA model are shown in **Figure 2C** and **Figure S3A**, respectively. One hundred permutation tests are shown in **Figure S3B**. Several pathways, including leukotriene metabolism, biopterin metabolism, tyrosine metabolism, valine, leucine, and isoleucine degradation, and prostaglandin formation from dihomogama-linoleic acid, were enriched **(** **Figure S3C** **)**. The discriminatory features were identified by MS/MS analysis. A total of 13 differential metabolites were identified, and 11 metabolites had potential diagnostic values with AUC values above 0.7 **(** **Table S2C**, **Table S2D** **)**. Using a logistic regression algorithm, tetrahydrocortisone, cortolone, and 18-carboxy dinor leukotriene B4 established a robust model for distinguishing between MB and benign samples, with an AUC value of 0.900 in the discovery group (**Figure S3D**, **Table 2**) and 0.873 in the validation group **(** **Figure 2D** **) (**sensitivity: 0.892 and specificity: 0.882).

Then, the difference between MB and other malignant brain tumors was also analyzed. PCA and OPLS-DA revealed the discrimination of the two groups, as shown in **Figure 2E** and **Figure S4A**, respectively. One hundred permutation tests showed the model stability (**Figure S4B** **)**. Pathway enrichment analysis showed significant enrichment pathways related to the carnitine shuttle, urea cycle/amino group metabolism, and fatty acid metabolism **(** **Figure S4C** **)**. Overall, 7 differential metabolites were identified (**Table S2E** **)**, and a total of 5 metabolites had diagnostic values with AUCs values above 0.7 **(** **Table S2F** **).** A panel consisting of L-Dopa, 20-oxo-leukotriene E4, cortolone, and tetrahydrocortisone showed predictive ability, with an AUC value of 0.842 in the discovery group **(** **Figure 2D**, **Table 2** **)** and 0.885 in the validation group **(** **Figure 2F** **) (**sensitivity: 1 and specificity: 0.702).

### Specific Urinary Metabolites of MB Patients

From the above three comparisons, 17 significantly different metabolites were discovered. Group clustering analysis of these 17 metabolites showed that the distance between MB and other malignant brain tumors was close, and the brain benign disease group was close to the healthy control. Functional annotation of metabolites showed that metabolites involved in leukotriene B, dopamine and steroid hormone biosynthesis were upregulated in MB patients, including cortolone, 20-oxo-leukotriene E4 and tetrahydrocortisone. However, metabolites involved in fatty acid oxidation and norepinephrine metabolism were downregulated in the MB group. In addition, the benign brain disease group showed specific metabolic characteristics, with metabolites involved in fatty acid oxidation and norepinephrine metabolism presenting with the highest intensity **(** **Figure 2G** **).**

Cases after surgery were enrolled to evaluate whether the identified differential metabolites were associated with tumor load. Differential metabolites associated with tumor load might serve as potential biomarkers for MB surgery monitoring. Therefore, we further examined the change trends of these 17 metabolites in postsurgery cases at approximately one week and one month postoperation (29 and 80 patients, respectively) to evaluate the biological association of the potential biomarkers with MB tumor load. The average intensity heatmap (**Figure S5**) showed that several metabolites, including cortolone, urothion, tetrahydrocortisone, 11-beta-hydroxyandrosterone-3-glucuronide, norepinephrine, N-acetylasparagine and isovalerylcarnitine recovered to normal levels one month postsurgery. These metabolites might probably be associated with tumor load and could be used for MB treatment monitoring. Metabolites of deoxyuridine, 5-nonenoylglycine, (-)-11-hydroxy-9,15,16-trioxooctadecanoic acid and 3-hydroxyoctanoyl carnitine showed no significant difference between pre- and postsurgery. These metabolites might not be associated with tumor load or have a chronic response that requires more time to recover.

**Figure 3:**
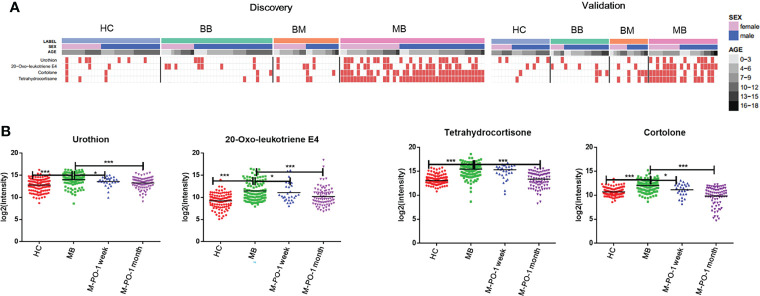
Relative intensity of MB-associated metabolites. The distribution of MB-associated metabolites in the discovery and validation groups **(A)**. The relative intensity of these 4 metabolites in the postoperative groups compared with the MB group **(B)**. “*”p < 0.05; “***”p < 0.001.

We expected better specificity for MB prediction using differential metabolites existing in all three comparisons. Four out of 17 metabolites, cortolone, urothion, 20-oxo-leukotriene E4, and tetrahydrocortisone, exhibited this property. The relative intensity of these four metabolites was plotted using a discrete heatmap plot. Taking the four metabolites with an intensity above the 90% quantile of the normal range in the healthy group as positively upregulated, they exhibited an upregulated prevalence in MB compared with the other three control groups in both the discovery and the validation groups (**Figure 3A** **).**

The urine levels of the above 4 specific metabolites in postsurgery cases were analyzed. As shown in **Figure 3B**, at one week postsurgery, 3 out of 4 specific metabolites exhibited significant differences between preoperative and one-week postoperative MB samples. All 4 metabolites showed a larger significant difference between preoperative and postoperative MB patients at one month. The levels of the 4 metabolites in postoperative MB patients at one month returned to the healthy state. These results further validated the biological relevance of these metabolites in MB, highlighting their potential value for MB diagnosis and monitoring.

**Figure 4:**
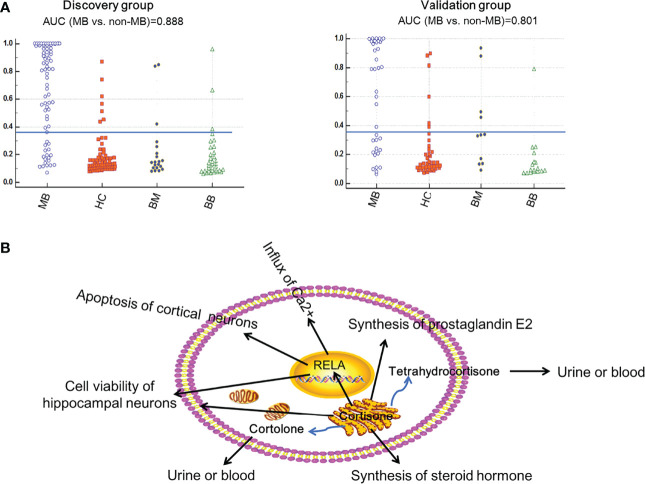
MB prediction using the metabolites tetrahydrocortisone and cortolone. The prediction accuracy of the metabolite panel (tetrahydrocortisone and cortolone) for MB in the discovery and validation groups **(A)**. The possible functions regulated by tetrahydrocortisone and cortolone in MB **(B)**.

The accuracy for distinguishing MB from non-MB using the 4 metabolites was assessed using ROC plots. They all showed AUC values above 0.7 for all three comparisons (**Table S2G** **)**. Finally, we found that the combination of cortolone and tetrahydrocortisone could distinguish MB from non-MB with better performance. The AUC value was 0.888 in the discovery group and 0.801 in the validation group **(** **Figure 4**, **Table 2** **)**. The identification of cortolone and tetrahydrocortisone was further validated by commercial standards (**Figure S6** **).**

## Discussion

The rapid development of high-throughput mass spectrometry has promoted urine metabolomics as a promising approach for the early detection of disease biomarkers. In this study, we comprehensively characterized the urine metabolomics of MB patients, including healthy subjects, patients with malignant brain tumors and patients with benign brain diseases. Potential biomarkers for MB were explored and tentatively identified.

In the present study, an altered metabolism, including fatty acid oxidation, steroid hormone biosynthesis, dopamine metabolism and leukotriene B4 metabolism, was discovered in MB patients when compared with healthy controls and patients with other brain diseases. Altered metabolism is a hallmark of tumor cells, which need to adapt to their nutrient-poor microenvironment to sustain their viability (18). Several studies have established that metabolism is altered in MB patients, including decreased fatty acid oxidation and increased lipogenesis (19), which is consistent with our results. Previous CSF metabolomics analysis suggests that a state of hypoxia might occur in MB patients, reflected by the accumulation of lipids and an increased TCA cycle (14). Additionally, CSF metabolomics analysis of MB patients discovered strongly upregulated lipid hormones (20). In the present study, upregulation of steroid hormones was shown in MB patients, indicating consistent metabolism changes in urine and CSF.

### Metabolomics Characteristics of MB Patients Compared With Other Diseases and Healthy Controls

The hierarchical cluster analysis results suggested that MB was more closely related to malignant brain tumors and showed large differences from healthy controls and benign brain diseases **(** **Figure 2G** **)**. These cluster results were consistent with the clinicopathological characteristics.

Compared to the other three groups, the MB group showed decreased levels of metabolites involved in fatty acid oxidation. Carnitine transports long-chain fatty acids into the mitochondrial matrix for beta-oxidation to provide cellular energy. Decreased acylcarnitine levels may be due to increased utilization of lipids or enhanced phospholipid and cholesterol synthesis, which is needed for increased membrane synthesis or formation of eicosanoids. Additionally, decreased concentrations may be a reflection of the role of carnitine and its acylesters in preserving the physiologic membrane structure and function from oxidative damage (21). In MB, neural progenitors metabolize glucose to lactate and prioritize lipid synthesis over fatty acid oxidation (22). The “metabolic transformation” is a hallmark of MB (19).

In MB, metabolites involved in steroid hormone biosynthesis, dopamine metabolism and leukotriene B4 metabolism were in higher abundance. The steroid metabolites tetrahydrocortisone and cortolone were upregulated in MB patients. These two steroids could be transformed to cortisol, which is a glucocorticoid hormone that plays an important role in steroid hormone biosynthesis (23). Previous research found that high concentrations of cortisol could inhibit DNA repair (24), which has been found during MB occurrence (25, 26).

Additionally, metabolites involved in leukotriene B4 metabolism showed higher levels in MB. Leukotrienes are pro-infiammatory mediators that can regulate the occurrence of brain cancer by increasing vascular permeability in brain tumors (27, 28). Leukotrienes are found at high levels in most inflammatory lesions and are involved in the physiological changes of the inflammatory process (29). High levels of leukotriene metabolites in MB probably indicated different inflammatory response mechanisms mainly mediated by leukotrienes between MB and other brain tumors.

### Potential Biomarkers of MB

In the present study, four metabolites were found to be significantly different between the MB and the other three groups. These 4 metabolites might be used as potential specific biomarkers of MB (detailed information in **Table S2G**).

Of those metabolites, tetrahydrocortisone and cortolone showed potential value in distinguishing MB from non-MB. Tetrahydrocortisone and cortolone are the downstream products of corticosterone (30). Corticosterone could regulate the function of the protein RELA, which is a vital protein that exists in MB (31). RELA activity is mainly associated with cell viability of hippocampal neurons (32), apoptosis of cortical neurons (33), influx of Ca^2+^ (34) and synthesis of prostaglandin E2 (35). Therefore, tetrahydrocortisone and cortolone might play an essential role in MB through the regulation of key signaling molecules (RELA) **(** **Figure S6** **)**. The detailed regulatory mechanisms need to be further investigated.

In the present study, MB-associated urinary metabolite changes were explored, and potential urinary biomarkers were tentatively discovered but need further deep validation. First, potential biomarker validation using targeted metabolomics is needed to quantify the absolute content of urinary metabolites. Second, due to the small sample size in this study, our evaluation is preliminary. Larger sample cohorts from multicenter analyses should be evaluated in the future for a more comprehensive validation. Third, the extent of resection might significantly vary from case to case. Individual variations could influence metabolite changes between the presurgery and control samples. Additionally, the samples collected before and after surgery were taken from different individuals. Individual variations could influence metabolite changes between presurgical and postsurgical groups. Therefore, dynamic monitoring of metabolite changes using a larger sample size collected from one case (pre- and postsurgery) is needed in future analyses.

## Conclusions

In this study, we utilized the LC–MS approach to define a panel of urine metabolites as a diagnostic biomarker for MB. The results suggested that the urine metabolome could differentiate patients with MB from healthy controls and other brain diseases. and can also reflect different disease states of the brain, both preoperatively and postoperatively. Furthermore, we identified two metabolites that exhibited good predictive ability for MB and non-MB discrimination, which could offer great value in clinical diagnosis and postoperative monitoring.

## Data Availability Statement

The datasets presented in this study can be found in online repositories (iProX - integrated Proteome resources). The names of the repository/repositories and accession number(s) is as following:URL: https://www.iprox.cn/page/PSV023.html;?url=1656564453623ejzW. PASSword: PdNB.

## Ethics Statement

The studies involving human participants were reviewed and approved by Institutional Review Board of the Institute of Basic Medical Sciences, Chinese Academy of Medical Sciences. Written informed consent to participate in this study was provided by the participants’ legal guardian/next of kin.

## Author Contributions

XL, JL, and XH conceived and designed the study, helped to interpret the data, wrote the first draft of the manuscript, and contributed to the final version of the manuscript. YZ, LJ and LZ collected the urine samples. HS performed mass analysis. YT helped the data analysis. WS and YT are the guarantor of this work and, as such, had full access to all the data in the study and takes responsibility for the integrity of the data and the accuracy of the data analysis. All authors contributed to the article and approved the submitted version.

## Funding

This work was supported by National Natural Science Foundation of China (No.82170524,31901039), Beijing Medical Research (No.2018-7), CAMS Innovation Fund for Medical Sciences (2021-1-I2M-016) and Biologic Medicine Information Center of China, National Scientific Data Sharing Platform for Population and Health.

## Conflict of Interest

The authors declare that the research was conducted in the absence of any commercial or financial relationships that could be construed as a potential conflict of interest.

## Publisher’s Note

All claims expressed in this article are solely those of the authors and do not necessarily represent those of their affiliated organizations, or those of the publisher, the editors and the reviewers. Any product that may be evaluated in this article, or claim that may be made by its manufacturer, is not guaranteed or endorsed by the publisher.

## Abbreviations

MB, medulloblastoma; LC-MS, liquid chromatography-mass spectrometry; QC, quality control; PCA, principal component analysis; OPLS-DA, orthogonal partial least squares discriminant analysis; GBM, glioblastoma multiforme.
