# Supplementary material for: LC–MS-Based Urine Metabolomics Analysis for the Diagnosis and Monitoring of Medulloblastoma
Source: Front Oncol. 2022 Jul 22;12:949513. doi: 10.3389/fonc.2022.949513 (PMC9353006; doi:10.3389/fonc.2022.949513)
Supplement: Supplementary file 1 [file DataSheet_1.pdf]

Fig. S1 Assessment of QC samples. (Trend plot showing the variation of t [1] over all QC Samples on urine metabolome.)

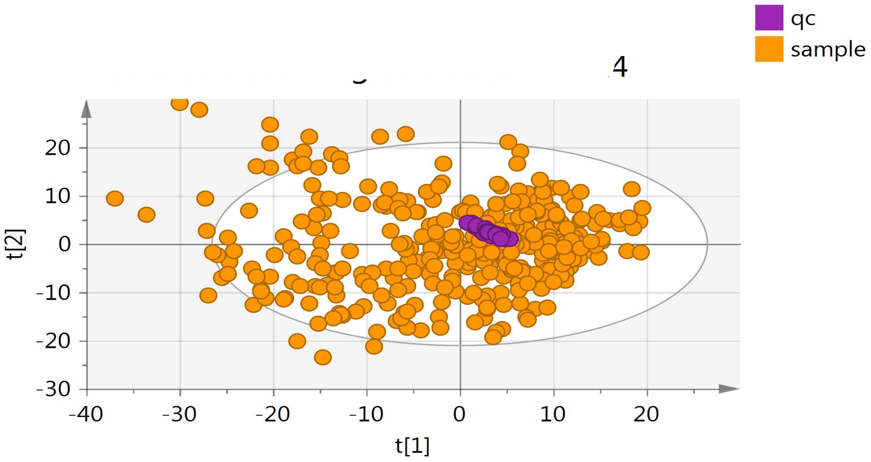

Fig. S2 Analysis of metabolic profiling variation of medulloblastoma and health control. (**a.** Score plot of supervised OPLS-DA overview of urine metabolic profiling between medulloblastoma and health control. **b.** One hundred permutation tests of the OPLS-DA model based on medulloblastoma and health control urine metabolome. **c.** Top five shifted metabolic pathways in medulloblastoma compared with health control. **d**. ROC plot with discovery group for distinction of medulloblastoma and health control based on tetrahydrocortisone, cortolone, N-acetylasparagine).

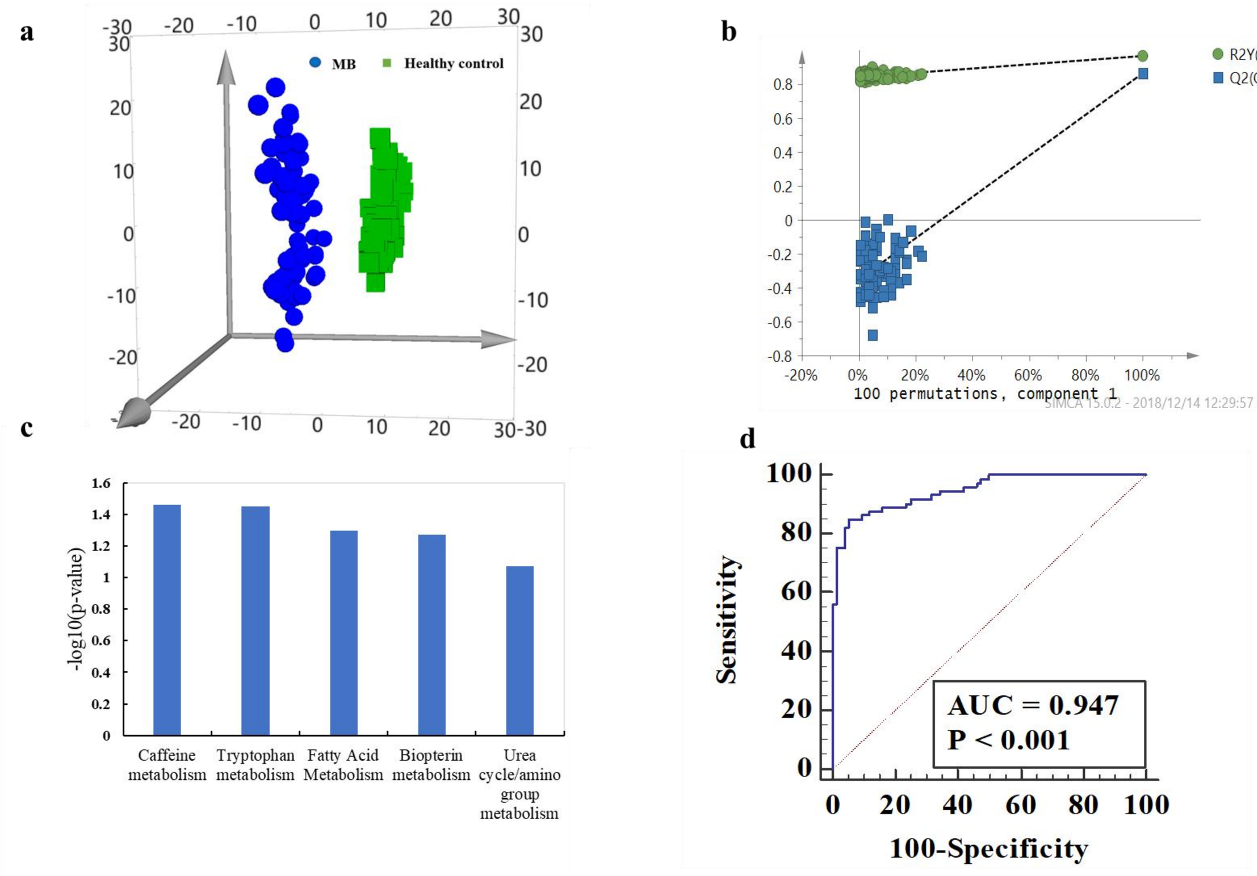

Fig. S3. Analysis of metabolic profiling variation of medulloblastoma and brain benign diseases. (**a**. Score plot of supervised OPLS-DA overview of urine metabolic profiling between medulloblastoma and brain benign diseases. **b**. One hundred permutation tests of the OPLS-DA model based on medulloblastoma and brain benign diseases urine metabolome. R2=(0.0,0.649) Q2=(0.0,-0.28) **c.** the metabolic pathway in medulloblastoma comparing with benign diseases. **d**. ROC plot with discovery group for distinction of medulloblastoma and brain benign diseases based on tetrahydrocortisone, cortolone,18-carboxy dinor Leukotriene B4.)

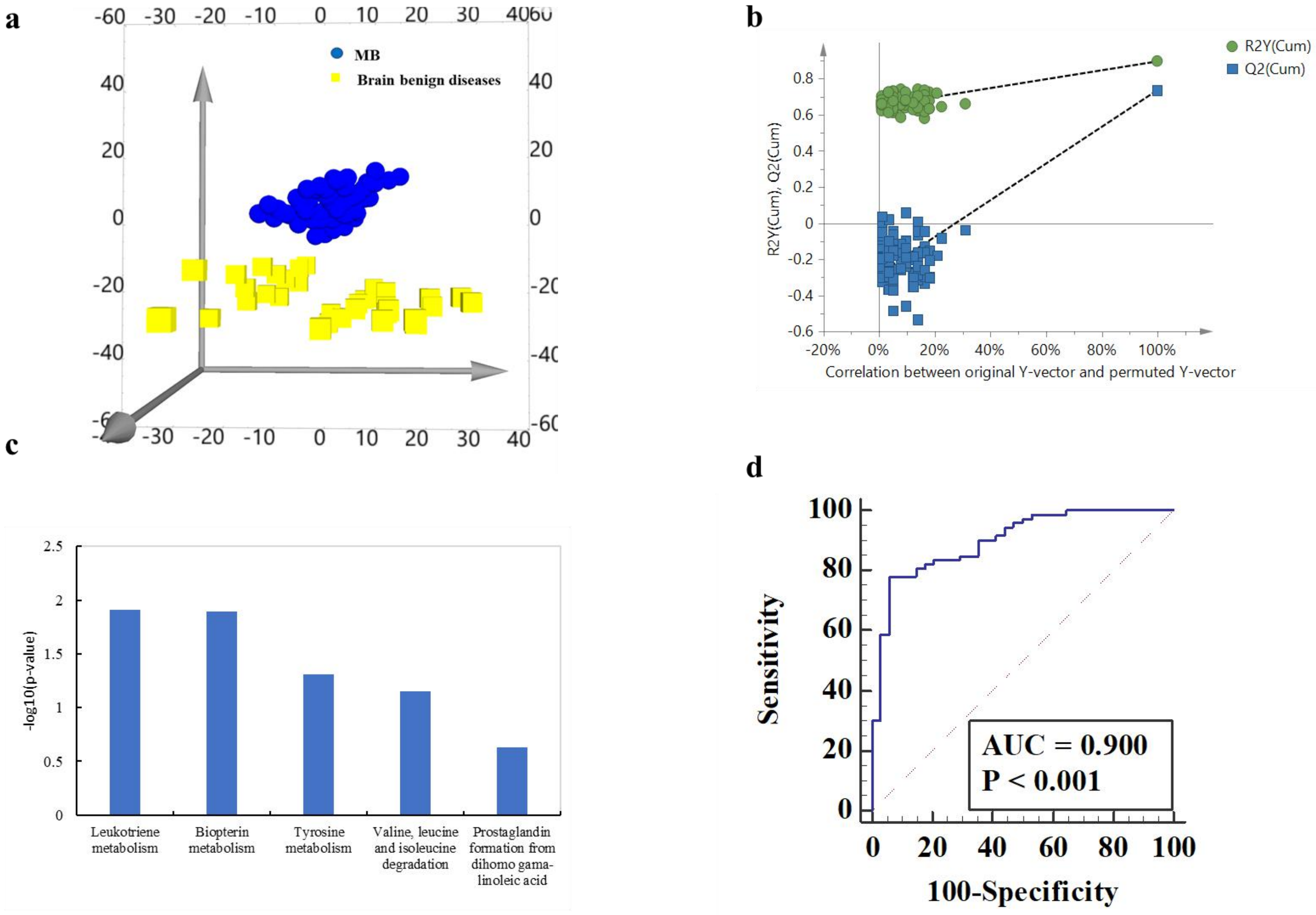

Fig. S4 Analysis of metabolic profiling variation of medulloblastoma and other brain malignant tumor. (**a.** Score plot of supervised OPLS-DA overview of urine metabolic profiling between medulloblastoma and other brain malignant tumor. **b.** One hundred permutation tests of the OPLS-DA model based on medulloblastoma and other brain malignant tumor urine metabolome. R2=(0.0,0.702) Q2=(0.0,-0.28) **c.** the metabolic pathway in medulloblastoma comparing with other brain malignant tumor. **d.** ROC plot with discovery group for distinction of medulloblastoma and other brain malignant diseases based on L-Dopa, 20-Oxo-leukotriene E4, cortolone, tetrahydrocortisone.)

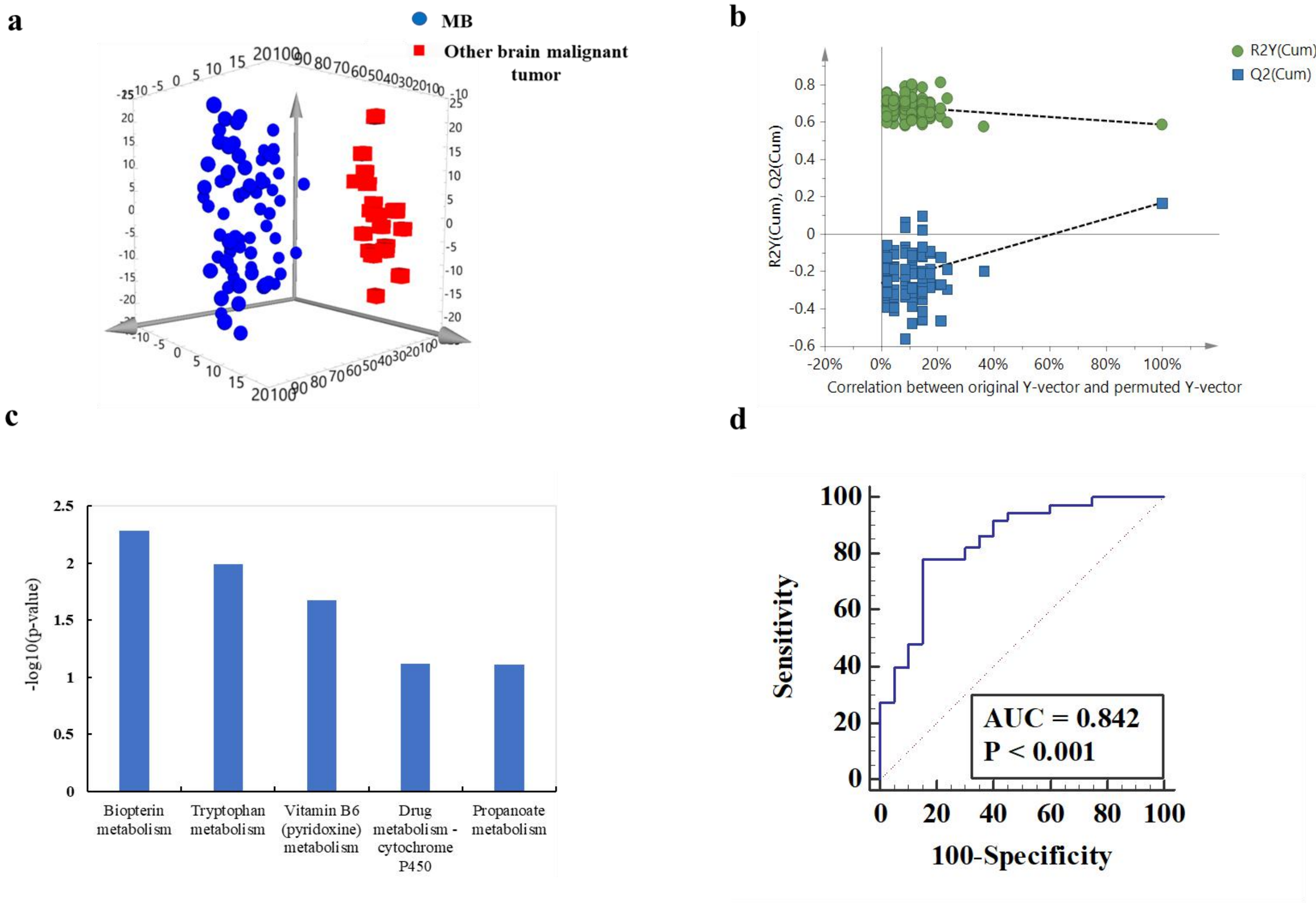

Fig. S5 The average intensity heatmap of the specific urinary metabolites of MB

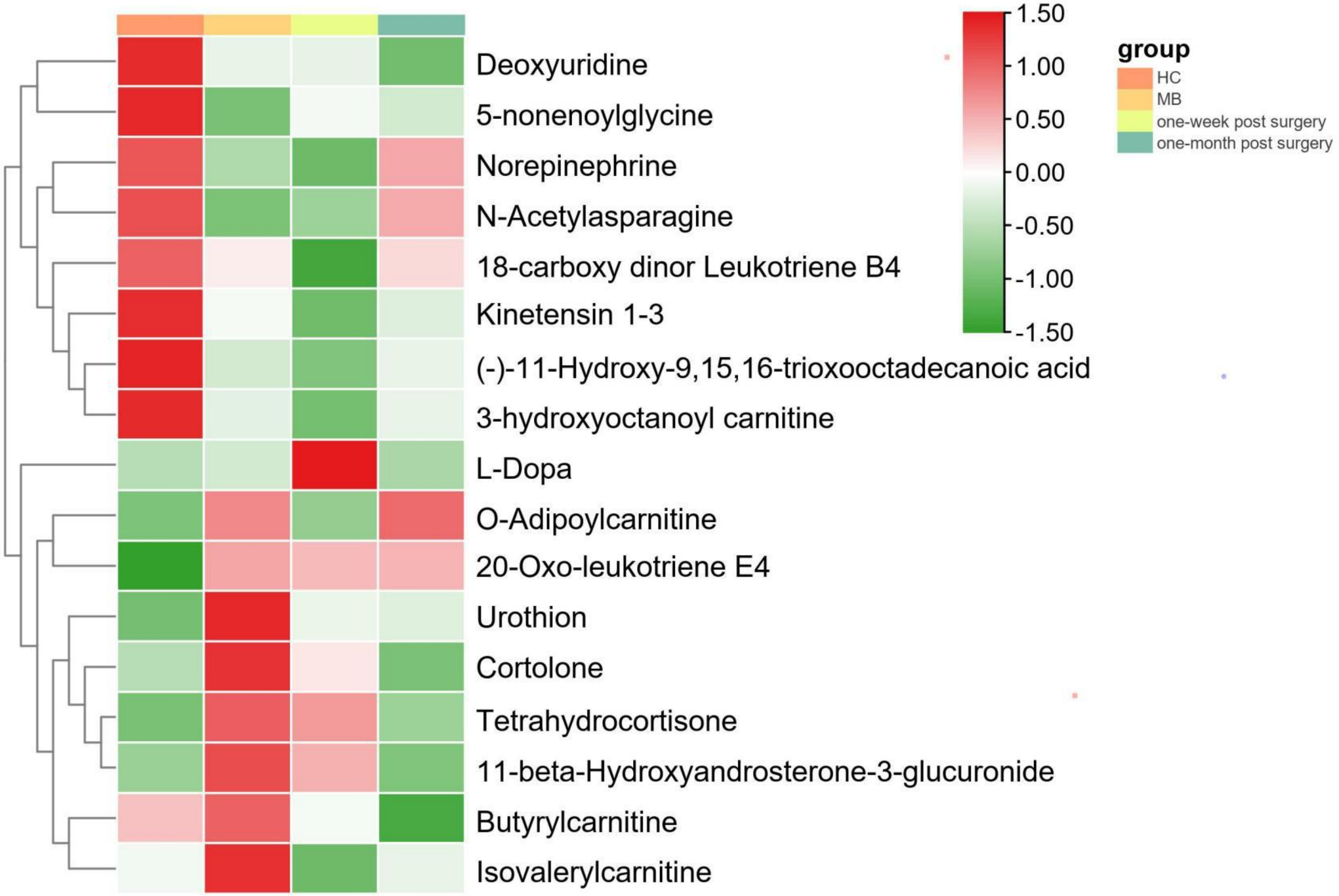

Fig S6. MS2 spectra of tetrahydrocortisone (a) and cortolone (b) in QC sample and standard compounds (STD) (a) tetrahydrocortisone 6.56_364.2237n 347.2204336

(1) QC

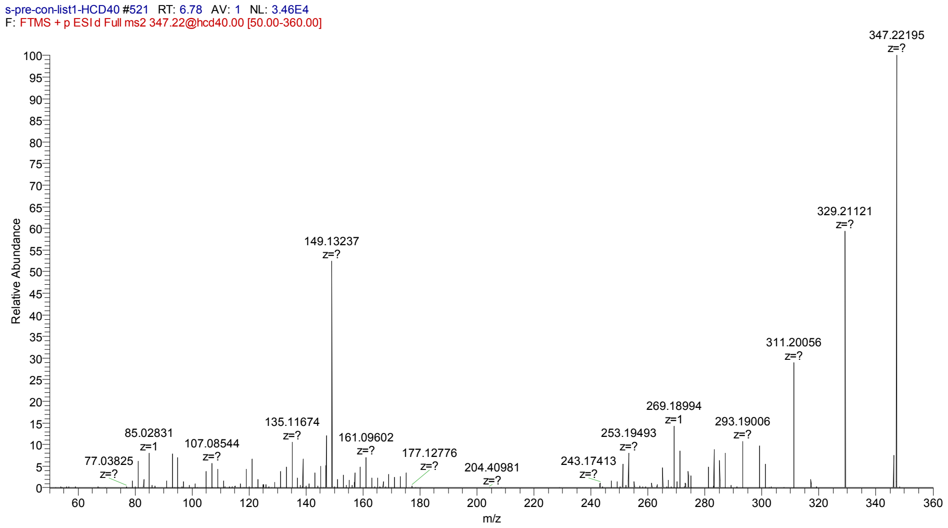

(2) STD

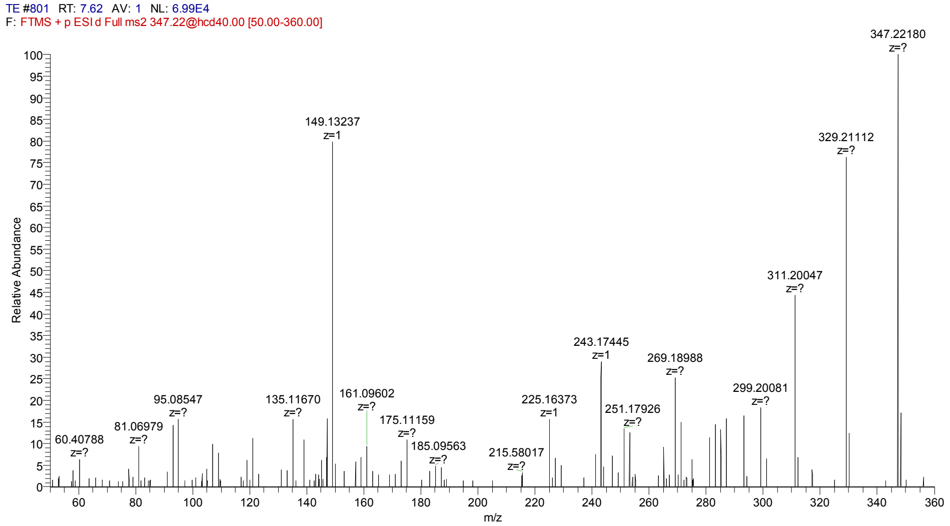

b）cortolone 6.54_349.2360m/z 349.2359908

(1) QC

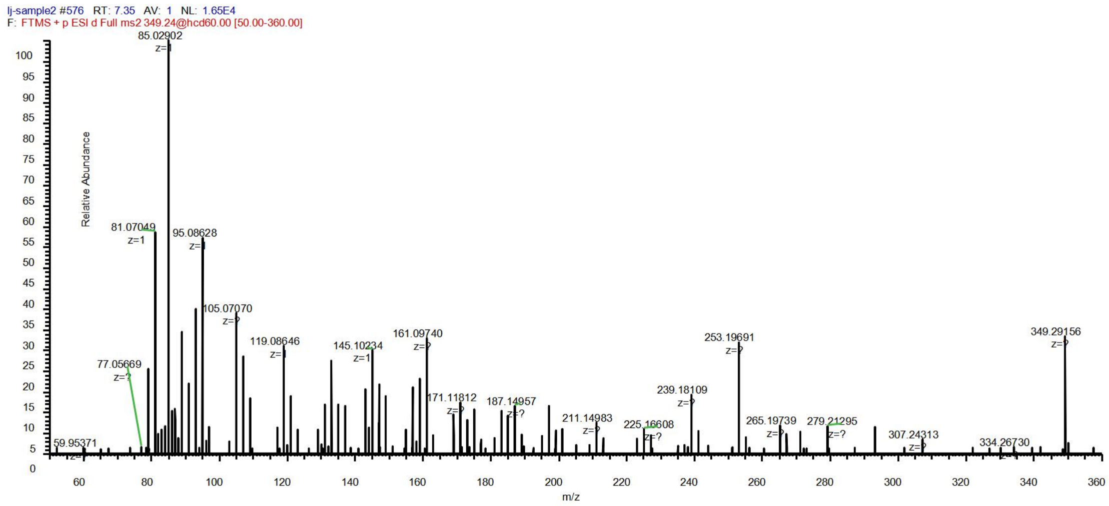

(2)STD

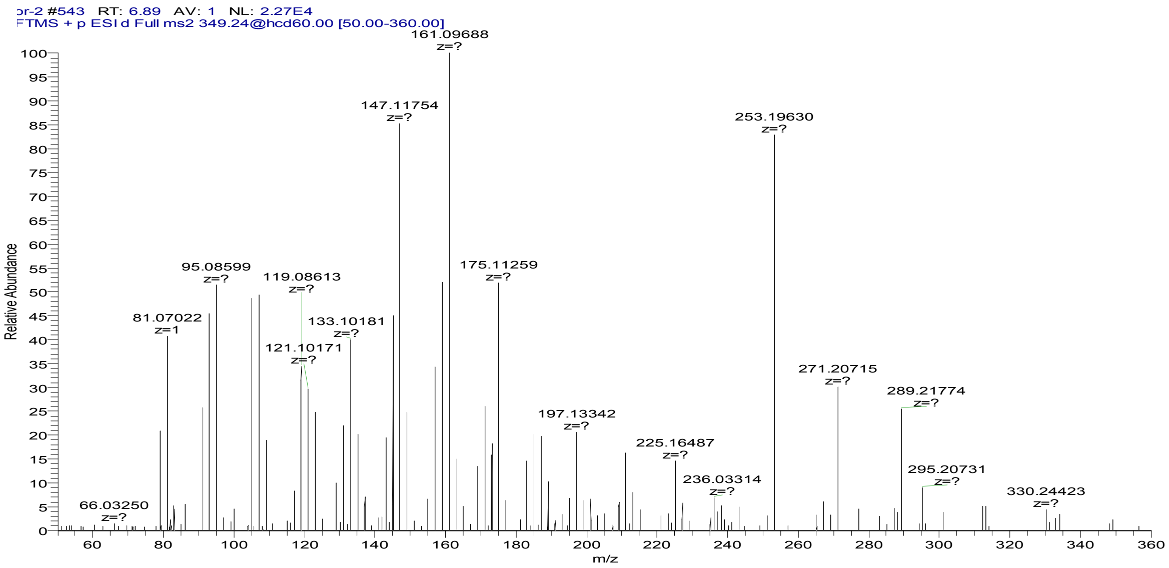

pr-2 #543 RT: 6.89 AV: 1 NL: 2.27E4
FTMS + p ESI d Full ms2 349.24@hcd60.00 [50.00-360.00]
161.09688 z=?
147.11754 z=?
253.19630 z=?
95.08599 z=?
119.08613 z=?
175.11259 z=?
81.07022 z=1
133.10181 z=?
121.10171 z=?
271.20715 z=?
289.21774 z=?
197.13342 z=?
225.16487 z=?
295.20731 z=?
236.03314 z=?
330.24423 z=?
66.03250 z=?
Relative Abundance
m/z
