# Supplementary material for: LC–MS-Based Urine Metabolomics Analysis for the Diagnosis and Monitoring of Medulloblastoma
Source: Front Oncol. 2022 Jul 22;12:949513. doi: 10.3389/fonc.2022.949513 (PMC9353006; doi:10.3389/fonc.2022.949513)
Supplement: Supplementary file 2 [file Presentation_1.pdf]

Supplemental Methods

LC-MS Analysis

Urinary metabolites were separated with a 17 min gradient on a Waters HSS C18 column (3.0× 100 mm, 1.7μm) at a flow rate of 0.3 mL/min. Mobile phase A was 0.1% formic acid in H2O and mobile phase B was acetonitrile. The gradient was set as follows: 0–1 min, 2% solvent B; 1 – 3min, 2 – 15% solvent B; 3 – 6 min, 15-50% solvent B; 6-9 min, 50-95% solvent B; 9-9.1 min, 95-100% solvent B; 9.1-12 min, 100% solvent B; 12-12.1min, 100-2% solvent B and 12.1-17min, 2% solvent B. Serum metabolites were separated with a gradient The column temperature was set as 50℃.

Full MS acquisition scanned from 100 to 1000 m/z at a resolution of 60 K. Automatic gain control (AGC) target was 1×10^6 and maximum injection time (IT) was 100 ms. UPLC targeted-MS/MS analyses were acquired at a resolution of 15 K with AGC target of 5×10^5, maximum IT of 50 ms, and isolation window of 3 m/z. Collision energy was optimized as 20, 40, 60 or 80 for each target with higher-energy collisional dissociation (HCD) fragmentation. The injection order of urine samples with 3 technical replicates was randomized to reduce the experimental bias.

Data processing using Progenesis QI

The detailed workflow for data processing facilitated by Progenesis QI is involved “create a new experiment”, “import data”, “review alignment”, “experiment design setup”, “peak picking”, “reviewed convolution--normalization”, and “identify compounds” in sequence. In general, the whole process ran automatically using optimized parameter settings. (1) In the stage of create a new experiment, adduct ion was carefully selected as it would influence the

number of characterized compounds and also the identification accuracy. Based on the ionization behaviors of reference standards, the adduct ion forms, comprising [M + H]+, [M + Na]+, [M + K], [M + NH4]+, [2M + H]+, [2M + Na]+, [2M + NH4]+, [M + H – H2O]+ and [M + H – 2H2O]+, were selected. (2) The MS data acquired by LC-MS for all the URINE samples were imported into the Progenesis QI software, generating a 2D ion intensity map with the retention time and m/z information as the ordinate and abscissa, respectively. (3) Peak alignment was carried out in automatic manner taking a QC run as the reference, the score values for all the samples were greater than 90 %. (4) For peak picking, the thresholds of chromatographic peak absolute intensity, and retention time limits can be set to achieve the maximum real ion signals with noise excluded. In the present study, absolute intensity and retention time limit were set at 1000 and default. "Normalize to all compound" was used to normalized peaks to eliminate sampling and analysis bias. (5) Further compound identification was performed by searching the HMDB database (2017 version). The identification results combined with the intensity data were exported as .csv files for subsequent compound confirmation and multivariate statistical analysis.

Confirmation of compounds characterization

Detailed compound identification information (.csv file) included compound ID, adducts, formula, score, fragmentation score, mass error (in ppm), isotope similarity, theoretical isotope distribution, web link, and m/z values. The data was further analyzed in detail, under which more abundant MS/MS fragments were acquired. Confirmation of the differential compounds was performed by the parameters, including Score, Fragmentation score, and Isotope similarity given by Progenesis QI. Score ranging from 0 to 60, is used to quantify the

reliability of each identity. According to the score results of the reference standards, the threshold was set at 35.0. Fragmentation score represents the matching degree between the theoretical fragments and the measured ones. The fragmentation score of 0 indicates no match occurs or the compound generates no fragments. Isotope similarity is calculated by comparison of the measured isotope distribution of a precursor ion with the theoretical. The compound identification is more reliable the higher the values obtained.

Statistical data analysis

Further data pre-processing including missing value estimation, log2 transformation and Pareto scaling were carried out to make features more comparable using MetaAnalyst 3.0 (http://www.metaboanalyst.ca). Variables missed in 50% or greater of all samples were removed from further statistical analysis. Non-parametric tests (Wilcoxon rank-sum test) were used to evaluate the significance of variables. False discovery rate (FDR) correction was used to estimate the chance of false positives and correct for multiple hypothesis testing. The adjusted p-value (FDR) cutoff was set as 0.05.

Metabolite annotation and pathway analysis

Mummichog is a program written in python for analyzing data from high－throughput, untargeted HRLC－MS metabolomics, bypassing the tedious and challenging metabolite identification. It leverages the organization of metabolic networks to predict functional pathways directly from feature tables and generate a list of tentative metabolites annotations through functional activity analysis. We input tab－delimited text files of peaks list with m/z, retain time, P value, and log2(FC) of two group analysis into Mummichog to conduct the pathways and module analysis. KEGG human network model was selected, and the cut－off

P value was set to 0.05 to generate a list of significant features. The analytical mode of mass spec was set to positive according the data source. Other options remained the default. Results from annotation, pathway analysis, and network module analysis were given. We then used MetaboAnalyst (http://www.metaboanalyst.ca/) to visualize the results files of the metabolic pathways network.
